# High Levels of Soluble P-selectin, Neutrophil Extracellular Traps, and Myeloperoxidase as Risk Factor of Deep Vein Thrombosis in Malignancy Patients Receiving Platinum-Based Chemotherapy

**DOI:** 10.12688/f1000research.146982.2

**Published:** 2025-07-17

**Authors:** Ni Made Renny Anggreni Rena, I Made Bakta, Ketut Suega

**Affiliations:** 1 Division of Hematology and Medical Oncology, Department of Internal Medicine, Faculty of Medicine, Udayana University, Denpasar, Bali, 80113, Indonesia

**Keywords:** Soluble P-Selectin, NET, MPO, risk factors, DVT

## Abstract

**Backgrounds:**

Venous Thromboembolism (VTE) is a disease entity comprising Deep Vein Thrombosis (DVT) and Pulmonary Embolism (PE). VTE events increase the mortality rate of patients with cancer receiving platinum-based chemotherapy. Soluble P-Selectin, Neutrophil Extracellular Traps (NET), and myeloperoxidase (MPO) are risk factors associated with DVT in malignancy patients receiving platinum-based chemotherapy. The purpose of this study was to determine the role of soluble P-selectin, NET, and MPO as risk factors for DVT in patients with malignancy receiving platinum-based chemotherapy.

**Patients and Methods:**

This study used a case-control design (matched pair case-control study) based on age and gender. The case group consisted of subjects with DVT, whereas the control group consisted of subjects without DVT. The subjects were 31 in each case and control groups. Soluble P-selectin, NET, and MPO levels were measured in each group.

**Results:**

The mean age of case group was 50.26±12.15 years meanwhile in control group was 52.81±11.64 years. In the case group, 71% of the subjects were female, whereas 51.6% of the control group were male. Most subjects, either in the case group (71%) or the control group (71%), used carboplatin. In the case group, cervix malignancy was the most common malignancy (32.3%), whereas in the control group, it was nasopharyngeal malignancy (25.8%). High soluble P-selectin level was a risk factor for DVT (OR 3.38, CI 1.180 – 9.780, p=0.02). A high NET level was also a risk factor for DVT (OR 2.88, CI 1.026-8.074, p=0.04). The high MPO levels in this study could not be proven as a risk factor.

**Conclusions:**

Soluble P-selectin and NET are risk factors that play a role in the pathophysiology of DVT through the pathomechanism of immunothrombosis induced by endothelial injury and activation of monocytes and neutrophils due to the use of platinum-based chemotherapy.

## Introduction

Venous thromboembolism (VTE) is characterized by Deep Vein Thrombosis (DVT) and Pulmonary Embolism (PE). Deep vein thrombosis (DVT) is a condition in which a thrombus forms in the venous system. Pulmonary embolism occurs when some of the thrombi released from deep vein thrombosis are trapped in the pulmonary circulation, thereby blocking blood flow to the lungs. ^1^^,^^2^ A high prevalence of VTE, a number of related risk factors, and high mortality from VTE are global concerns. Data obtained from several studies in Western Europe, North America, Australia, and Argentina have recorded the incidence of VTE per year ranging between 1.75 and 2.69 per 1,000 individuals. Based on the IDENTIA study in 2016, which nationally recorded the incidence of Deep Venous Thrombosis (DVT) in Indonesia, the data showed that the incidence in the medically ill population was quite high at 37.1%. ^2^

Venous thromboembolism (VTE) is one of the main causes of morbidity and mortality in patients with malignancies. Approximately 20% of all VTE patients with either deep vein thrombosis (DVT) or pulmonary embolism (PE) have malignancy as the underlying disease. The risk of VTE in patients with cancer is estimated to be 5-7.5 times greater than that in the general population. ^1^^,^^3^^,^^4^ Risk factors for VTE in patients with cancer can be grouped into three categories: patient-related factors (intrinsic and extrinsic), treatment-related factors, and cancer-related factors. The risk factors for VTE in cancer patients are the contributions of the three categories of risk factors, and the VTE risk caused by each risk factor is difficult to evaluate separately from one another. ^5^

Cancer patients receiving chemotherapy have a six-fold increased risk of VTE compared with the general population. A study by Heit JA, *et al.* in 2005 the odds ratio was 6.5 and 4.1 for the occurrence of VTE in cancer patients receiving chemotherapy and cancer patients not receiving chemotherapy. ^5^^–^^7^ Chemotherapy can induce a prothrombotic state through several mechanisms, including damage to the vascular endothelium by increasing the levels of procoagulant molecules and reducing endogenous anticoagulants. Chemotherapy can also induce tumor and endothelial cell apoptosis and cytokine release, leading to increased Tissue Factor (TF) expression and activity. Furthermore, chemotherapy triggers platelet activation and directly induces the expression of monocyte-macrophage TF. ^8^

Platinum-based chemotherapy is a chemotherapeutic drug used to treat several types of malignancies. Platinum-based chemotherapy is widely used, covering about half of the patients undergoing chemotherapy. ^9^ Platinum chemotherapy is a class of chemotherapy which is an inorganic compound that works through DNA binding and interferes with DNA repair mechanisms. In a retrospective review of VTE in patients with germ cell cancer treated with cisplatin and bleomycin-based chemotherapy, the risk of thrombosis was estimated to be 8.4%. A prospective evaluation of VTE in patients with non-small cell lung cancer (NSCLC) treated with cisplatin and gemcitabine showed a VTE incidence of 17.6%. ^8^

The mechanism underlying platinum-class chemotherapy drugs to increase thrombosis is thought to involve several mechanisms, such as decreased protein C activity and increased von Willebrand factor. A separate in vitro investigation revealed that cisplatin induces platelet activation. Elevated von Willebrand factor (vWF) levels suggest endothelial injury, and are associated with cisplatin-induced arterial thrombosis. ^8^

In addition, cisplatin can disrupt the balance of the cytokine profile by increasing Tumor Necrosis Factor (TNF), which is a procoagulant for endothelial cells and reduces prostacyclin synthesis, causing intravascular platelet aggregation. Endovascular damage occurs directly through free radical-induced lipid peroxidation in endothelial cells. This can lead to the thickening of the intimal tunica and platelet aggregation. in vitro evidence has shown that pharmacological doses of cisplatin increase TF activity in human blood monocytes without increasing TF expression. ^10^

Cancer cells have the capacity to express specific adhesion molecules that allow for attachment to blood vessel walls and interactions with blood cells as well as activation of the procoagulant properties of stem cells, especially endothelial cells, leukocytes, and platelets. The adhesion of tumor cells to endothelial cells significantly initiates clotting in the vessel wall and subsequent thrombus formation. Several adhesion molecules have been described to have a role in the adhesion of various types of tumor cells to endothelial cells, such as P-selectin. ^11^ Activated endothelial cells and platelets express P-selectin, which binds to cancer cells. P-selectin mediates binding to specific carbohydrate-containing ligands, such as P-selectin glycoprotein ligand-1, which is present on leukocytes and platelets. Thus, P-selectin supports the initial tethering of leukocytes to endothelial cells and activated platelets and mediates leukocyte attachment to endothelial cell surfaces. ^11^

Chemotherapy also causes an increase in tissue factors and activates monocytes and macrophages, which in turn activate neutrophils. Neutrophil Extracellular Traps (NET), a histone net of DNA and proteases produced by activated neutrophils, are one of the markers of thrombosis. Its role in both venous and arterial thromboses has been demonstrated in several animal studies. Neutrophil Extracellular Traps (NETs) play a role in inducing cell interaction and activation, resulting in thrombosis. Histones from NET can activate endothelial cells and increase the release of von Willebrand factor (a glycoprotein important for platelet adhesion and aggregation). In 2018, Mauracher *et al.* ^12^ found that increased histone citrullinated H3 (a biomarker for NET formation) was associated with an increased incidence of VTE in patients with cancer, whereas other NET biomarkers (DNA and nucleosomes) were associated with a higher risk of VTE during the first 3–6 months. These data demonstrate the importance of NET in the pathogenesis of cancer-associated thromboses. ^3^

Myeloperoxidase (MPO) is a heme protein secreted from activated neutrophils, monocytes, and macrophages, which mediates lipid peroxidation due to reactive processes.

Neutrophil activation, degranulation, and release of MPO have been reported in arterial thrombosis, such as unstable angina and acute myocardial infarction, owing to their suspected proatherogenic nature. Therefore, MPO has been suggested as a potentially useful marker for the initial assessment of patients with acute coronary syndromes. While the role of MPO in VTE cases has not been widely revealed, several studies have reported that neutrophil-binding MPO was detected in venous thrombus on immunohistochemical examination. ^13^

Due to the high incidence of VTE and mortality in malignancy patients receiving chemotherapy, platinum is one of the most widely used chemotherapy classes in clinical practice, and is closely related to the incidence of VTE. It is very important to know the risk factors and mechanisms underlying the occurrence of VTE. Several risk factors, such as P-selectin, NETs, and myeloperoxidase, are thought to play a role. These three factors are the result of the immune system's response to inflammation, including malignancy, so that based on VTE risk factors they are classified as patient-related intrinsic factors. Although some literature suggests that NETs are part of the interface between host immunity and cancer biology. ^14, 15^ This encourages researchers to determine whether P-selectin, NET, and MPO play a role as risk factors for VTE, especially DVT, in patients with malignancies who receive platinum-based chemotherapy. This study aimed to determine the role of soluble P-Selectin, Neutrophil Extracellular Traps (NET), and myeloperoxidase (MPO) in the occurrence of Deep Vein Thrombosis, particularly as risk factors in patients with malignancy receiving platinum-based chemotherapy.

## Methods

**Figure 1.**
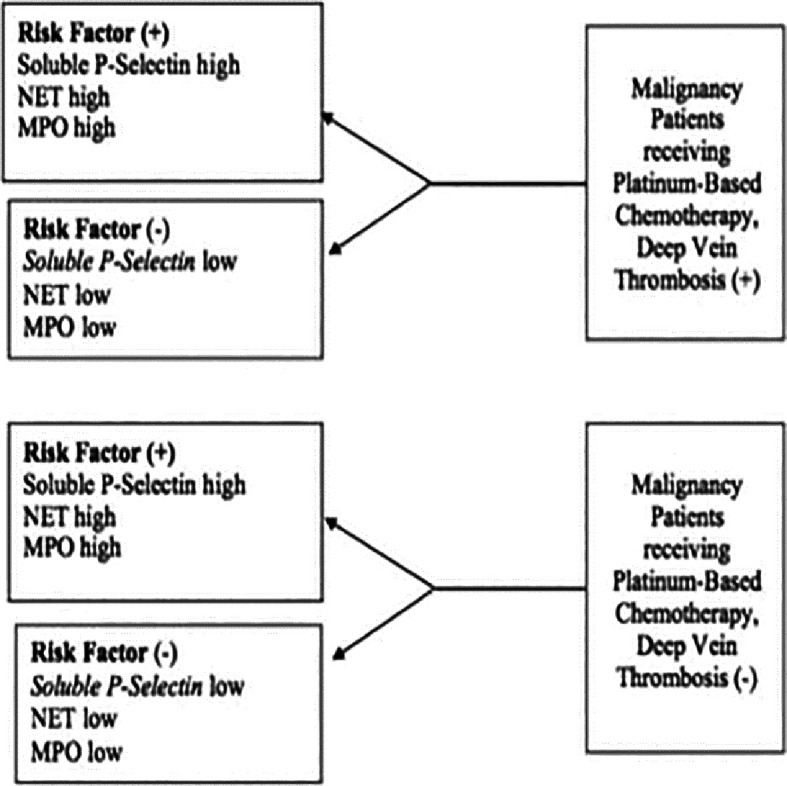
Research design.

This study used a case-control design (matched pair case-control study) based on age and gender. The study was conducted at the Polyclinic and Internal Medicine ward of Sanglah General Hospital Denpasar, which was conducted in December 2020 until the number of samples was met. Samples in the case group were cancer patients who received platinum-based chemotherapy, were treated at Sanglah General Hospital Denpasar, and experienced DVT. The control group included patients with cancer who received platinum-based chemotherapy and did not experience DVT. In each case and control group, 31 research participants were obtained by purposive sampling. Serum blood examination was performed to determine Soluble P-Selectin, NET, and MPO levels. Purposive sampling was performed on all patients with malignancy who received platinum-based chemotherapy at Sanglah Hospital who met the inclusion criteria and did not meet the exclusion criteria until the completion of the study. The explanation of the research design can be seen in Figure 1.

We then explain the benefits and objectives of the study, as well as the risks that may be experienced. If the patient voluntarily stated that they were willing to participate in the study, they were asked to sign an informed consent form. The informed consent given to research participants was written consent. This research has been ethically approved on 19 November 2020 by the Research Ethics Committee (KEP) Faculty of Medicine, Udayana University/Sanglah General Hospital Denpasar (No. 2303/UN14.2.2.VII.14/LT/2020).

Data on the questionnaire were collected through anamnesis, physical examination, and from the patient’s medical records, which included demographic data of the patient as well as previous medical history, surgery history, examination of vital signs, height and weight, general physical examination, signs of sepsis, heart defects, kidney disease, pregnancy, history of thrombosis, and liver disorders.

**Figure 2.**
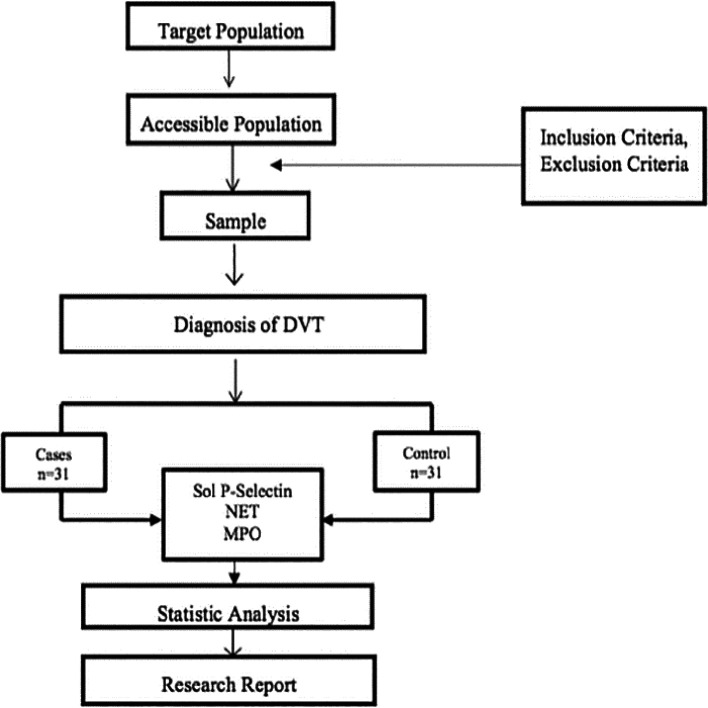
Research protocol.

Samples were determined for the case and control groups using the DVT diagnosis algorithm. The levels of Soluble P Selectin, NET, and MPO were measured in the blood. P-Selectin measurement used Human Soluble P-Selectin/CD62P Immunoassay while NET and MSO measurement used ELISA. All data obtained were statistically analyzed. Descriptive statistical analysis was used to describe the characteristics of the research subjects and the frequency distribution of several variables in this study, such as age, gender, type of malignancy, stage of malignancy, type of chemotherapy used, Well’s score, comorbid hypertension, and diabetes mellitus. Variables that scale numerical data and are normally distributed are displayed using mean and standard deviation. Categorical variables were presented as numbers and percentages. The results of the statistical analysis were presented in the form of distribution tables. Normality tests were performed using the Shapiro–Wilk test, and the data were normally distributed. To calculate the odds ratio of MPO levels, soluble P-Selectin and NET to Deep Vein Thrombosis using the Chi Square test based on a 2 × 2 table. This study also conducted multivariate tests on variables including confounding variables. The research protocol can be seen in Figure 2.

## Results

### Characteristics of research subjects by case and control group

This study included 62 participants. Both the case and control groups included patients with malignancy who received platinum-based chemotherapy. The case group consisted of 31 patients with malignancy who received platinum-based chemotherapy and had deep vein thrombosis (DVT), while the control group consisted of 31 study subjects with malignancy who received platinum-based chemotherapy without deep vein thrombosis (DVT). The characteristics of the study participants are presented in Table 1.

**Table 1.** Characteristics of subjects based on case and control groups.

Variable	Case (n = 31)	Control (n = 31)	p-value
**Mean age (years) ± SD**	50.26 ± 12.15	52.81 ± 11.64	0.72
**Gender**			
Male	8 (25.8%)	16 (51.6%)	0.07
Female	23 (74.2%)	15 (48.4%)	
**Stadium**			
Non-metastatic	22 (71%)	19 (61.3%)	0.53
Metastatic	9 (29%)	12 (38.7%)	
**Type of chemotherapy**			
Cisplatin	3 (9.7%)	8 (25.8%)	0.054
Carboplatin	22 (71%)	22 (71%)	
Oxaliplatin	6 (19.4%)	1 (3.2%)	
**Well’s score**			
1	21 (67.7%)	25 (80.6%)	0.45
2	8 (25.8%)	6 (19.4%)	
3	1 (3.2%)	0(0%)	
4	1 (3.2%)	0 (0%)	
**Hypertension**			
Yes	2 (6.5%)	4 (12.9%)	0.67
No	29 (93.5%)	27 (87.1)	
**Diabetes mellitus**			
Yes	1 (3.2%)	2 (6.5%)	0.55
No	30 (96.8%)	29 (93.5%)	

* Statistically significant at p < 0.05.

Regarding the type of malignancy recorded in this study, the majority of cases were cervical, colorectal, or ovarian malignancies. The control group had nasopharyngeal and cervical malignancies ( Table 2). Most of the samples during the study had non-metastatic clinical stages, both in the case and control groups. This is because patients with metastatic stage were mostly not administered chemotherapy due to their performance status; therefore, patients with metastatic stage were not recruited in this study.

**Table 2.** Types of malignancy research subjects based on case and control groups.

Type of malignancy	Group		p-value
	Case	Control	
	(n = 31)	(n = 31)	
**Colorectal**	6 (19.4%)	1 (3.2%)	0.038 *
**Cervix**	10 (32.3%)	6 (19.4%)	
**Ovarian**	5 (16.1%)	0 (0%)	
**Nasopharynx**	1 (3.2%)	8 (25.8%)	
**Breast**	2 (6.5%)	3 (9.7%)	
**Lung**	2 (6.5%)	3 (9.7%)	
**Lymphoma**	1 (3.2%)	2 (6.5%)	
**Bone**	0 (0%)	1 (3.2%)	
**Penis**	1 (3.2%)	1 (3.2%)	
**Others**	3 (9.7%)	6 (19.4%)	

* Statistically significant at p < 0.05.

### Results of soluble P-Selectin, Neutrophil Extracellular Traps (NET), Myeloperoxidase (MPO) levels

**Figure 3.**
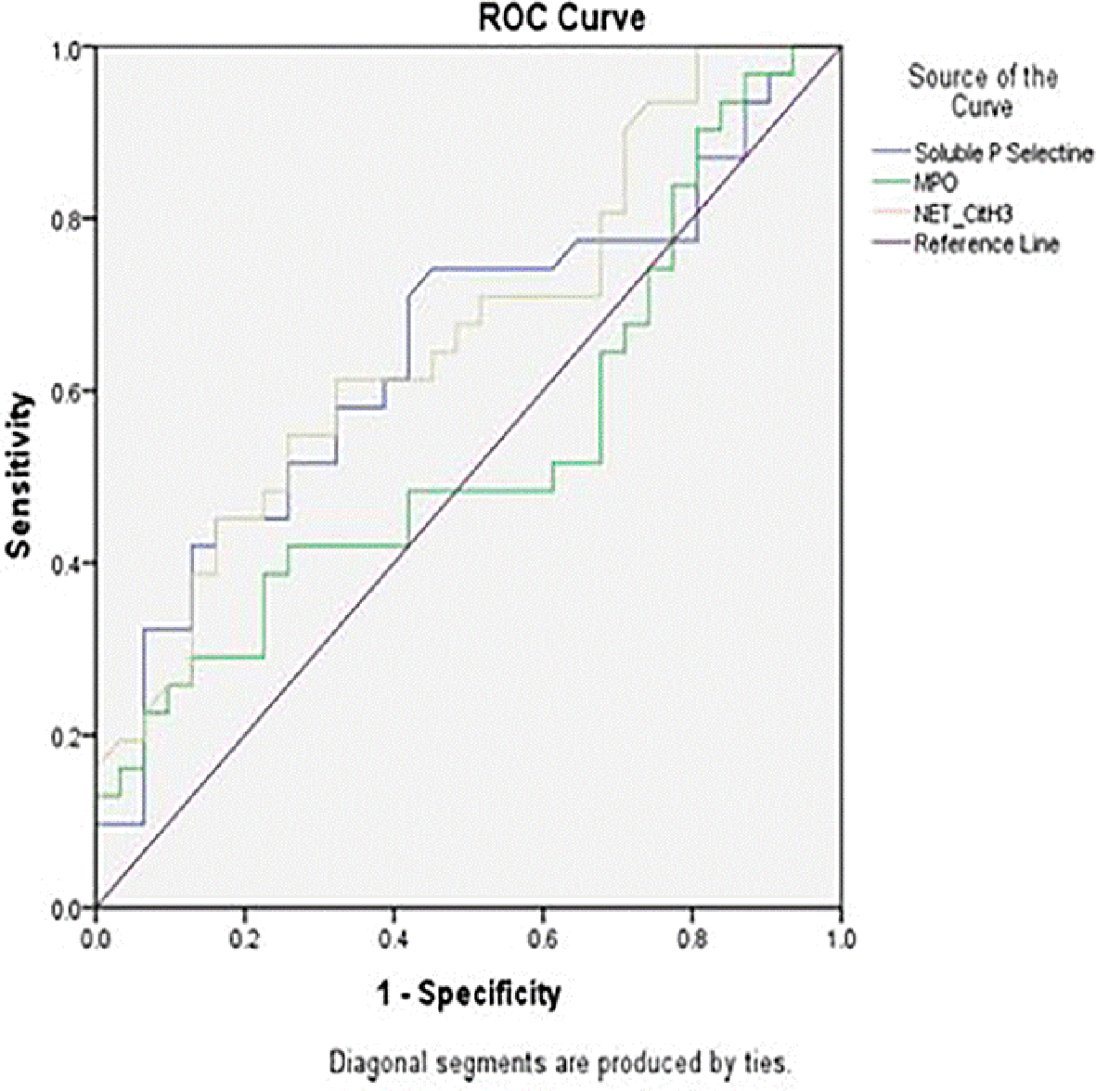
Area under curve ROC (Receiver Operating Characteristic) levels of Soluble P-Selectin, Neutrophil Extracellular Traps (NET) and Myeloperoxidase (MPO).

After examining the levels of Soluble P-Selectin, Neutrophil Extracellular Traps (NET) and Myeloperoxidase (MPO) in this study, then based on the Area Under Curve ROC (Receiver Operating Characteristic) as shown in Figure 3, the cut-off point of the three variables was obtained and grouped the data into two categories, high and low. Based on the ROC curve, the cut-off value used for Soluble P-Selectin was 82.84 ng/mL. The sensitivity and specificity of Soluble P-Selectin were 0.57 (95% CI = 0.30– 0.82, p < 0.001) and 0.73 (95% CI = 0.51–0.90, p < 0.001) and the Diagnostic Odds Ratio (DOR) was 4.31 (95% CI = 2.22–8.37, p < 0.01). The ROC curve showed significant accuracy of Soluble P-Selectin (AUC = 0.74, p = 0.05). For NET, a cut-off value of 5.89 was obtained with an AUC of 0.66, sensitivity of 0.61, and specificity of 0.64. Meanwhile, the cut-off value for MPO was 644.14 mg/mL, with an AUC of 0.54, sensitivity of 0.42, and specificity of 0.74.

The results of the examination of the levels of Soluble P Selectin, neutrophil extracellular traps (NET), and myeloperoxidase in this study were grouped based on the cut-off points obtained as shown in Table 3.

**Table 3.** The results of the examination of the levels of Soluble P Selectin, Neutrophil Extracellular Traps (NET) and Myeloperoxidase.

Type of malignancy	Group		Odds Ratio	95% Confidence Interval	p-value
	Case	Control			
	(n = 31)	(n = 31)			
**Category of Soluble P-Selectin (ng/mL)**					
High (≥82.84)	22 (71%)	13 (41.9%)	3.38	1.180 – 9.780	0.02
Low (<82.84)	9 (29%)	18 (58.1%)			
**Category of NET (ng/mL)**					
High (≥5.89)	19 (61.3%)	11 (35.5%)	2.88	1.026 – 8.074	0.04
Low (<5.89)	12 (38.7%)	20 (64.5%)			
**Category of MPO (ng/mL)**					
High (≥644.14)	13 (41.9%)	8 (25.8%)	2.08	0.709 – 6.085	0.18
Low (<644.14)	18 (58.1%)	23 (74.2%)			

* Statistically significant at p < 0.05.

Based on Table 3, it was found that MPO did not have a significant difference between the case and control groups. This study also explored MPO as a continuous variable into its relationship with DVT that potentially reveals associations missed by categorizing it as high/low. The results of the MPO analysis as continuous data can be seen in Table 4. The data show that MPO has no significant impact on DVT even as numeric data (p = 0.588).

**Table 4.** Results of MPO analysis as continuous data on DVT.

Variable	Case	Control	Mean diff	Normality test	p-value
MPO	528.7 ± 212.1	505.2 ± 206.5	23.53	0.012	0.588 *

* Non-parametric test with Mann-Whitney test.

This study was then continued by conducting a multivariate analysis to assess variables including confounding variables to DVT. This study found that both Soluble P Selectin, Neutrophil Extracellular Traps, NET, MPO, and confounding variables such as gender, type of chemotherapy, and type of cancer did not have a significant impact on the incidence of DVT (p > 0.05). The results of multivariate analysis can be seen in Table 5.

**Table 5.** Results of multivariate analysis of the levels of soluble P Selectin, NET, MPO, and confounding variables on DVT.

Variable	OR	95%CI	P-value
Soluble P Selectin	0.314	0.088 – 1.114	0.073
NET	0.709	0.187 – 2.691	0.613
MPO	0.457	0.109 – 1.918	0.285
Gender	0.450	0.115 – 1.759	0.251
Type of Chemotherapy	0.379	0.081 – 1.774	0.218
Cancer Type	1.158	0.874 – 1.535	0.306

* Statistically significant at p < 0.05.

## Discussion

Based on sex characteristics, the proportion of female subjects (74.2%) was higher than that of males (25.8%) in the case group, while in the control group, there were more males (51.6%). However, the sex difference between the two groups was not significant. A previous study in Japan found that female compared to males had a 2.32 times higher risk for VTE than men. ^16^ A multicentre, prospective, observational registry study in 12 hospitals throughout Indonesia, including Sanglah Hospital Denpasar, showed that 54.19% of the sample was female and the remaining 45.81% were male. ^2^ In this study, females were found more in the case group, related to the underlying malignancy, which is ovarian malignancy, cervical malignancy, and breast malignancy. Although few studies have investigated the effect of sex on the incidence of VTE and the relationship between sex and the location of VTE, a study conducted by Barco *et al.* in 2018 stated that even sex can have an impact on the characteristics that show symptoms and prognosis in patients with VTE. ^17^

Regarding the age limit of the research subjects, this study used adult patients (aged > 18 years) with an age range of 20–71 years. The mean ages of the two groups were not significantly different. In the case group, it was found with 50.26 ± 12.15 years, while in the control group, the mean age was 52.81 ± 11.64 years. Theoretically, advancing age increases the risk of developing VTE, and some studies have found that people over 60 years of age have a higher risk of developing VTE compared to those under 60 years of age. ^5^ In this study, several samples obtained were patients with malignancies at a young age, such as osteosarcoma, cervical cancer, and Hodgkin’s lymphoma; therefore, the mean age of this study was less than 60 years.

Regarding the type of malignancy recorded in this study, the majority of cases were cervical, colorectal, or ovarian malignancies. The control group had nasopharyngeal and cervical malignancies ( Table 2). Most of the samples during the study had non-metastatic clinical stages, both in the case and control groups. This is because patients with metastatic stage were mostly not administered chemotherapy due to their performance status; therefore, patients with metastatic stage were not recruited in this study.

Cancer is associated with an increased risk of VTE, which in turn is associated with an increased risk of death in patients with all stages and types of cancer. Recent research on approximately one million cancer patients recorded in the University Health System Consortium database showed that 41,666 (4.1%) cancer patients experienced VTE in the hospital. A large observational cohort study showed that the types of cancer associated with the highest VTE rates were pancreatic, brain, ovarian, uterine, kidney, stomach, colon, rectal, and lung cancers. In these patients, the cancer sites associated with the highest incidence of VTE were the pancreas (8.1%), ovaries (5.6%), kidneys (5.6%), lungs (5.1%), stomach (4.9%), and brain (4.7%). The incidence of VTE is also high in patients with hematology malignancies such as leukemia, Hodgkin’s disease, non-Hodgkin’s lymphoma, and myeloma, with VTE rates ranging from 4.2% to 5.0%. ^5^^,^^18^

Based on the data on the type of chemotherapy used by patients in this study, both in the DVT group and without DVT, most patients used carboplatin. A total, 71% used carboplatin chemotherapy, 19.4% used oxaliplatin, and 9.7% used cisplatin. In the group without DVT, 71% used carboplatin, 25.8% used cisplatin, and 3.2% used oxaliplatin. There was no significant difference between the two groups (p = 0.054). This retrospective study was conducted to estimate the risk of VTE development in patients receiving cisplatin chemotherapy. A total of 200 samples were treated with cisplatin chemotherapy and 200 samples were treated without cisplatin chemotherapy. The basic characteristics of the two study groups were similar. The results of this study showed that the incidence of VTE in the Cisplatin group was 2.8 times higher than that in the non-cisplatin group (95% CI 1.4 – 4.2). ^19^

### Soluble P-Selectin as a risk factor for deep vein thrombosis

The results of this study support several previous studies regarding the role of Soluble P-Selectin in cancer patients as a risk predictor and as a diagnostic tool to detect the presence of VTE. Previous study showed 167 (93.82%) patients were suspected of having DVT and after a diagnostic examination, it was confirmed that 59 (35.33%) patients had DVT and 11 (6.18%) had pulmonary embolism. There was a significant difference in the mean levels of Soluble P-Selectin in this study (p < 0.001). ^5^

A study by Setiawan *et al.* found that 12.5% ​​of patients experienced DVT. His study found that patients who experienced DVT had significant soluble P-selectin and von Willebrand factor. Soluble P-selectin concentrations were also significantly increased after chemotherapy (81.95 ng/mL vs 92.5 ng/mL, p < 0.001). The study found that both platelets and endothelial cells appear to be activated by chemotherapy. ^20^ The study by Vandy *et al.* reported that in a study conducted on all inpatients and outpatients for 3 years, 159 patients with confirmed DVT had statistically significant levels of Soluble P Selectin, D-dimer, CRP, and von Willebrand factor. In this study, the Soluble P-Selectin cut-off point used was > 90 ng/mL combined with Wells Score 2 as a DVT diagnostic tool with a specificity of 96%, positive predictive value of 89%, and negative predictive value of 77%. ^21^

A study conducted by Antonopaulus *et al.* found that Soluble P-Selectin levels increased after the occurrence of VTE (OR = 2.89, 95% CI = 2.31–3.61, p < 0.001) or DVT only (OR = 2.64, 95% CI = 1.95-3.56, P < 0.001). This study excluded patients with solid tumors and HIV-positive patients with lupus anticoagulants. The aggregated sensitivity and specificity of Soluble P-Selectin were 0.57 (95% CI = 0.30–082, p < 0.001) and 0.73 (95% CI = 0.51-0.90, p < 0.001) and the Diagnostic Odds Ratio (DOR) 4.31 (95% CI = 2.22–8.37, p < 0.01). The ROC curve showed significant Soluble P-Selectin accuracy (AUC = 0.74, p = 0.05). This indicates that Soluble P-Selectin is significantly increased in patients with DVT, with or without PE complications, with high diagnostic performance. ^22^

Another study showed that there was a significant difference in Soluble P-Selectin levels between patients in the case and control groups, with a cut-off point of 70.5 ng/ml with a sensitivity of 98%, specificity of 100%, NPV of 96.8%, and PPV of 100%. ^22^ Platinum-based chemotherapy induces thrombosis through several mechanisms including direct vascular endothelial damage, induction of cell apoptosis, and subsequent production of microparticles. In addition, platinum-based chemotherapy is also suspected to directly induce the activation of monocytes and macrophages that produce Tissue Factor (TF), and is able to induce an increase in von Willebrand factor. ^8^ P-Selectin is one of a cell adhesion molecule (CAM) found on the surface of activated endothelial cells, which coat the inner surface of blood vessels and activated platelets. Soluble P-selectin is the soluble form of P-selectin in the circulation and is considered to be a key molecule in haemostasis and mediating the inflammatory process in thrombosis. Soluble P-selectin induces the production of procoagulant microparticles and increases fibrin deposition. ^23^ Soluble P-selectin and P Selectin Glycoprotein Ligan-1 (PSGL-1) play a role in the interaction of platelets with neutrophils in the innate immune system and in the formation of Neutrophil Extracellular Traps (NET), known as NETosis, an important process in the immuno-thrombosis system.

Theoretically, when viewed from the perspective of the Virchow Triad (stasis, endothelial injury, and hypercoagulability), in this study, an increase in Soluble P-Selectin levels in malignancy patients reflects a hypercoagulable (prothrombotic) condition, and the use of chemotherapy results in endothelial injury, thereby increasing the risk of thrombosis in cancer patients. ^5^ The results of this study prove the hypothesis that high Soluble P-Selectin levels in malignancy patients using platinum-based chemotherapy can be used as a risk factor for DVT.

### Neutrophil Extracellular Traps (NET) as a risk factor for deep vein thrombosis

The administration of platinum-based chemotherapy to cancer patients increases the risk of developing DVT. Through several mechanisms that have been described, platinum-based chemotherapy mostly induces the process of thrombosis formation through endothelial injury and increased expression of tissue factors, thereby increasing activated monocytes and neutrophils as well as through platelet activation mechanism, thereby inducing the emergence of a NETosis process so that NET is formed.

Neutrophil extracellular traps (NETs) are composed mostly of chromatin and neutrophil granular proteins such as neutrophil elastase (NE), myeloperoxidase (MPO), and cathepsin G. The results of several studies have indicated that neutrophils are a major component of the intravascular immune response to circulating blood pathogens. As host protection against pathogens, neutrophils secrete neutrophil extracellular traps (NETs). In addition to the capacity of intracellular mechanisms to fight infection, neutrophils also utilize extracellular devices to protect the host from cellular damage caused by infection. Several studies have suggested a role for NETs in tumor progression, metastasis, and cancer-associated thrombosis. ^24^^,^^25^

The role of NET in the mechanism of immune-thrombosis can occur through several pathways, such as direct activation of factor XII and binding to Von Willebrand factor in recruiting and activating more platelets. In addition, the enzymes contained in NET, such as neutrophil elastase (NE) and myeloperoxidase (MPO), are able to cause inactivation of endogenous anticoagulants, such as Tissue Factor Pathway Inhibitor (TFPI), and NET is able to bind directly to tissue factors and activate the extrinsic haemostasis system. The activation of intravascular blood coagulation and thrombus formation in microvessels (microvascular thrombosis) under certain conditions that support this intravascular immune mechanism is called immunothrombosis. ^24^

The main player in the release of neutrophils from the chromatin nucleus, known as neutrophil extracellular traps (NET), is citrullinated histone H3 (H3Cit). The increase in plasma H3Cit reported in several studies was found to be significant in thrombotic patients compared to that in healthy individuals. Neutrophil analysis in thrombotic patients showed a greater proportion of H3Cit intracellular neutrophils than in healthy individuals. The presence of plasma H3Cit in thrombotic patients correlates strongly with the activation of the markers of neutrophil elastase (NE), myeloperoxidase (MPO), and the inflammatory cytokines IL-6 and IL-8, known as NETosis. H3Cit can be detected in the nuclei of neutrophils at the time of stimulation and released into the blood circulation at the time of NETosis. ^26^

Thalin *et al.* suggested that H3Cit is a potential blood plasma marker for the diagnosis and prognosis of thrombosis and cancer. Examination of H3Cit in the circulating blood is crucial for diagnosis and prognosis. ^26^ In this study, the authors used the H3Cit marker to represent NET produced in patients with malignancy undergoing platinum-based chemotherapy. The results of this study are similar to those of other studies that have been carried out, such as the cohort study by Mauracher *et al.*, who found that high plasma NET levels, identified by the H3Cit biomarker, are a fairly specific prognostic indicator of an increased risk of VTE 2 years after diagnosis or recurrence in cancer patients. An increase in H3Cit to 100 ng/mL is known to increase the risk of VTE by 13%. ^12^

In a cohort study followed for 2 years, it was found that cancer patients had elevated NET biomarkers, and 2.3% of them had arterial thromboembolism (ATE). An increase in H3Cit levels was also associated with an increased risk of developing ATE. This study also found that increasing H3Cit up to 100 ng/mL is known to increase the hazard ratio by 1.1 higher for mortality in patients with cancer. ^27^

### Myeloperoxidase (MPO) as a risk factor for deep vein thrombosis

The studies were grouped into high and low, with a cut-off point of 644.14 ng/mL. High MPO levels were observed between the case and control groups with an Odds Ratio of 2.08 with a 95% Confidence Interval of 0.709 – 6.085. Myeloperoxidase (MPO) is a peroxidase enzyme that is synthesized and stored in the cytoplasm of neutrophils. MPO is mostly expressed in immune cells, such as leukocytes, polymorphonuclear neutrophils, lymphocytes, monocytes, and macrophages. MPO is secreted under certain conditions such as chronic inflammation, infection, and other conditions that involve the immune system. Activated neutrophils, monocytes, and macrophages release MPO at sites of inflammation. ^23^ Previous study examining the relationship between plasma DNA (including MPO) and DVT biomarkers (Von Willebrand Factor (VWF)), D-dimer, and P-selectin found a unidirectional relationship between plasma DNA levels compared to other DVT biomarkers. ^28^ In the pathogenesis of VTE, it is known that one is caused by damage to the vascular endothelium, causing the induction of tissue factor and F.VII; thus, venous thrombosis is associated with the inflammatory process. Research using a murine model supports this by demonstrating the contribution of platelets, monocytes, and neutrophils to the process of thrombus initiation and propagation. Several recent studies have also shown a contribution to the formation of Neutrophil Extracellular Traps (NET) in venous thrombosis, and MPO is suspected that MPO is a component of NET. ^26^ After inflammation due to vascular endothelial damage, endothelial cells secrete massive amounts of Von Willebrand Factor (VWF) and P-selectin. Secretion of these two components enhances platelet adhesion and leukocyte withdrawal and induces NET production. In addition, the production of cytokines by endothelial cells (IL-1β, IL-8, and ROS) accelerates NET formation. ^29^

Another mechanism underlying the relationship between MPO and VTE is the increased consumption of Nitric Oxide (NO) that occurs as a result of the inflammatory process. MPO itself binds to NO in the process, resulting in an increase in NO consumption both through the inflammatory process and by MPO itself. This condition is thought to cause inflammation of the blood vessel endothelial cells, which worsens. ^29^

MPO is a biomarker of neutrophil activation and is involved in the pathogenesis of immune-thrombosis. Neutrophil activation, degranulation, and markers of MPO release are now widely used as biomarkers for unstable angina and acute myocardial infarction, which are thought to be proatherogenic and play a role in destabilizing atherosclerotic plaques. MPO is widely recommended as an early biomarker for patients with acute coronary syndrome. Venous thromboembolism (VTE) is a condition associated with excessive free radical production in a homeostatic system that has not shown much efficiency. ^29^

This study showed that high MPO levels are not proven to be a risk factor for DVT in malignancy patients receiving platinum-based chemotherapy. One factor that can affect the results of the examination of MPO levels is that the MPO substrate is the same as the general peroxidase substrate. Hemoglobin is known to exhibits pseudoperoxidase activity, which can interfere with assays measuring true peroxidase enzymes such as myeloperoxidase (MPO). Hemoglobin’s intrinsic peroxidase-like activity may contribute to background signal or assay interference, potentially leading to underestimation or overestimation of MPO levels. ^30, 31^ Based on the ELISA kit, there are no further cross-reactivity data in human samples, but the kit states that recombinant mouse MPO cross-reacts at approximately 0.4% while neutrophil elastase cross-reacts at approximately 0.25%. However, in this study, we did not assess and analyze hemoglobin levels in both case and control groups. We acknowledge this as a limitation and will consider reviewing and adjusting for hemoglobin levels in future studies to determine whether MPO retains prognostic relevance when hemoglobin-related interference is accounted for. Additionally, myeloperoxidase can be detected by cytometry, immunohistochemistry, or cytochemical staining. Some literature states that from existing studies comparing different levels of myeloperoxidase tests have not obtained satisfactory results, so further standardization and validation are needed. ^29, 31^

The relationship between various types of cancer and Soluble P Selectin, NET, and MPO levels is currently still being developed. P-selectin interacts with various cancer types through its ligands on tumor cells. Previous studies document the binding of P-selectin to various cancer-derived cell lines, which shows its role in facilitating metastatic behavior and cancer cell adhesion. However, specific statements regarding the effect of cancer types on soluble P-selectin have not been further studied. ^32, 33^ Meanwhile, NET generated from activated neutrophils have been implicated in many types of cancer progression both solid tumor and hematological malignancy. The specific studies on the direct correlation between NETs and cancer type are also limited. On the other hand, the presence of MPO as inflammatory mediators and markers is often increased in cancerous conditions. Increased MPO levels in cancer patients are thought to correlate with disease activity and poor prognosis, especially among myeloid malignancies and solid tumors. This study focuses on how Soluble P Selectin, NET, and MPO affect DVT in platinum-based chemotherapy with various types of cancer. There is not much information about the type of cancer that can affect these levels. ^34^ Therefore, further research is expected to assess the effect of cancer type on Soluble P Selectin, NET, and MPO levels.

One factor that can affect the results of the examination of MPO levels is that the MPO substrate is the same as the general peroxidase substrate. Additionally, myoglobin and haemoglobin are known to exhibit peroxidase activity, which may impair the results. Myeloperoxidase can be detected by cytometry, immunohistochemistry, or cytochemical staining. Some literature states that from existing studies comparing different levels of myeloperoxidase tests have not obtained satisfactory results, so further standardization and validation are needed. ^29^ The results of myeloperoxidase (MPO) examination in this study were grouped into high and low, with a cut-off point of 644.14 ng/mL. High MPO levels between the case and control groups were not proven to be a risk factor for DVT in malignancy patients receiving platinum-based chemotherapy. ^13^

Therefore, Soluble P-Selectin and Neutrophil Extracellular Traps (NET) play a role in the pathophysiology of Deep Vein Thrombosis (DVT) in malignant patients who receive platinum-based chemotherapy. The administration of anticoagulants as VTE thromboprophylaxis can be considered according to risk stratification based on existing recommendations. The pathomechanism is thought to be an immune thrombosis process, which is a new concept that is being developed. The presence of endothelial injury and activation of monocytes and neutrophils due to platinum-based chemotherapy induces the formation of NETosis, which is the initial initiation of thrombosis.

## Ethics and consent

This research has been ethically approved on 19 November 2020 by the Research Ethics Committee (KEP) Faculty of Medicine, Udayana University/Sanglah General Hospital Denpasar (No. 2303/UN14.2.2.VII.14/LT/2020).

## Data Availability

Figshare: Data Tabulation.doc, https://doi.org/10.6084/m9.figshare.25398304.v1. ^35^ Figshare: Informed Consent.docx, https://doi.org/10.6084/m9.figshare.25398298. ^36^ Figshare: Data Collection Form.doc, https://doi.org/10.6084/m9.figshare.25398301.v1. ^37^ Figshare: PROCEDURE AND METHODS (1).doc, https://doi.org/10.6084/m9.figshare.25398307.v1. ^38^ Data are available under the terms of the Creative Commons Attribution 4.0 International license (CC-BY 4.0).
