# Supplementary material for: High Levels of Soluble P-selectin, Neutrophil Extracellular Traps, and Myeloperoxidase as Risk Factor of Deep Vein Thrombosis in Malignancy Patients Receiving Platinum-Based Chemotherapy
Source: F1000Res. 2025 Jul 17;13:427. Originally published 2024 Apr 30. [Version 2] doi: 10.12688/f1000research.146982.2 (PMC11729192; doi:10.12688/f1000research.146982.2)
Supplement: Peer review history

# Reviewer report: Reviewer response for version 2

**DOI:** 10.5256/f1000research.183975.r398937

**Published:** 2026-01-03

**Authors:** Irawan Cosphiadi

**Affiliations:** 1 Division of Hematology and Medical Oncology, Department of Internal Medicine, Faculty of Medicine Universitas Indonesia-Cipto Mangunkusumo National General Hospital, Universitas Indonesia, Jakarta, Indonesia

The authors have done the revisions

Is the work clearly and accurately presented and does it cite the current literature?

Yes

If applicable, is the statistical analysis and its interpretation appropriate?

Yes

Are all the source data underlying the results available to ensure full reproducibility?

Yes

Is the study design appropriate and is the work technically sound?

Yes

Are the conclusions drawn adequately supported by the results?

Yes

Are sufficient details of methods and analysis provided to allow replication by others?

Partly

Reviewer Expertise:

Hematology and medical oncology

I confirm that I have read this submission and believe that I have an appropriate level of expertise to confirm that it is of an acceptable scientific standard.

# Reviewer report: Reviewer response for version 2

**DOI:** 10.5256/f1000research.183975.r398935

**Published:** 2025-11-03

**Authors:** Shinta Wardani

**Affiliations:** 1 Division of Hematology and Oncology, Department of Internal Medicine, Faculty of Medicine, Universitas Brawijaya, Malang, Indonesia

To Ni Made Renny Anggraeni Rena 

 I appreciate you taking the time to incorporate this feedback so comprehensively.

 I am **satisfied with all the revisions** and agree with your rationale:

- The classification of **soluble P-selectin, NETs, and MPO** as patient-related intrinsic factors is clearly justified in the Introduction.
- The addition of literature regarding the **influence of cancer type** successfully contextualizes your findings.
- Your explicit acknowledgment of the **hemoglobin level** limitation, along with the potential for MPO assay interference, is a valuable and transparent addition to the Limitations section.

These changes have significantly enhanced the clarity and robustness of your manuscript

 Best Regards

 Shinta Oktya Wardhani

Is the work clearly and accurately presented and does it cite the current literature?

Yes

If applicable, is the statistical analysis and its interpretation appropriate?

I cannot comment. A qualified statistician is required.

Are all the source data underlying the results available to ensure full reproducibility?

Yes

Is the study design appropriate and is the work technically sound?

Yes

Are the conclusions drawn adequately supported by the results?

Yes

Are sufficient details of methods and analysis provided to allow replication by others?

Yes

Reviewer Expertise:

NA

I confirm that I have read this submission and believe that I have an appropriate level of expertise to confirm that it is of an acceptable scientific standard.

# Reviewer report: Reviewer response for version 2

**DOI:** 10.5256/f1000research.183975.r398936

**Published:** 2025-08-21

**Authors:** Eko Adhi Pangarsa

**Affiliations:** 1 Diponegoro University, Dr. Kariadi General Hospital, Semarang, Indonesia

Accepted.

Is the work clearly and accurately presented and does it cite the current literature?

Partly

If applicable, is the statistical analysis and its interpretation appropriate?

Yes

Are all the source data underlying the results available to ensure full reproducibility?

Yes

Is the study design appropriate and is the work technically sound?

Yes

Are the conclusions drawn adequately supported by the results?

Partly

Are sufficient details of methods and analysis provided to allow replication by others?

Yes

Reviewer Expertise:

Medical Oncology, Hematology (B cell malignancy), Thrombosis Hemostasis

I confirm that I have read this submission and believe that I have an appropriate level of expertise to confirm that it is of an acceptable scientific standard.

# Reviewer report: Reviewer response for version 1

**DOI:** 10.5256/f1000research.161114.r276428

**Published:** 2025-01-13

**Authors:** Shinta Wardani

**Affiliations:** 1 Division of Hematology and Oncology, Department of Internal Medicine, Faculty of Medicine, Universitas Brawijaya, Malang, Indonesia

This is my short summary report of my Review.

 This article addresses the link between Soluble P-selectin, neutrophil extracellular trap (NETs) and Myeloperoxidase (MPO) as risk factors of Deep Vein Thrombosis in malignancy patients receiving platinum based that would be an important contribution for our data since this is the first data for Indonesian patients.

 There are a number of issues that need to be clarified :

 1. In introduction mentions that VTE risk factors in patients with cancer can be grouped into three categories :

- Patient-related factor (intrinsic and extrinsic)
- Treatment-related factors and
- Cancer related factors

    but it has not been explained in detail whether the position of soluble P-selectin, NETs and MPO is a patient-related factor, treatment-related factor or cancer related 

     factor. If it is a patient-related factor it could be a novelty data specific for Indonesian people.

 2. In this study the most common type in the case group was Cervical cancer and in the control group dominated by Nasopharynx cancer. I needs authors opinion about 

     These different types of cancer do not affect the level of P-selectin, NETs and MPO. 

     If differences in cancer type affect the levels of these three biomarkers maybe the author could give a brief additional information as your research limitation.

 3. In the last paragraph of page 10 noted that haemoglobin level is known to exhibit peroxidase activity which may impair the results of MPO level and might be the 

     reason why MPO level was not proven to be a risk factor for DVT in your study result. Perhaps researchers can review the research data to see if there is a difference 

     in mean haemoglobin between case and control group which can explain that the difference haemoglobin is a factor causing the insignificance of MPO results as a 

     risk factor for DVT

 Since only need minor revision I suggest to ACCEPT this article.

Is the work clearly and accurately presented and does it cite the current literature?

Yes

If applicable, is the statistical analysis and its interpretation appropriate?

I cannot comment. A qualified statistician is required.

Are all the source data underlying the results available to ensure full reproducibility?

Yes

Is the study design appropriate and is the work technically sound?

Yes

Are the conclusions drawn adequately supported by the results?

Yes

Are sufficient details of methods and analysis provided to allow replication by others?

Yes

Reviewer Expertise:

Hematologi and Medical Oncology

I confirm that I have read this submission and believe that I have an appropriate level of expertise to confirm that it is of an acceptable scientific standard.

# Response

**Published:** 2025-07-05

**Authors:** Ni Made Renny Anggreni Rena

**Affiliations:** Internal Medicine, Udayana University, Jimbaran, Bali, Indonesia

Thank you very much for your constructive feedback. We have added your feedback in the manuscript:

 - We found that soluble P-selectin, NETs and MPO are patient-related factor. These three factors are the result of the immune system's response to inflammation, including malignancy, so that based on VTE risk factors they are classified as patient-related intrinsic factors. Although some literature suggests that NETs are part of the interface between host immunity and cancer biology. We have added this explanation to the introduction section in the last paragraph.

 - We have added several studies that discussed the influence of cancer type on the levels of P-selectin, NETs and MPO in paragraph 5 of sub-chapter 'Myeloperoxidase (MPO) as a risk factor for deep vein thrombosis'. Until now, there has been no research data that mentions the components of thrombosis molecule levels in each cancer that can affect Soluble P Selectin, NET, and MPO levels. 

 - In this study, we did not assess and analyze hemoglobin levels in both case and control groups. We acknowledge this as a limitation and will consider reviewing and adjusting for hemoglobin levels in future studies but we found other study stated that hemoglobin’s intrinsic peroxidase-like activity may contribute to background signal or assay interference, potentially leading to underestimation or overestimation of MPO levels.

 We sincerely acknowledge your constructive input, which has enhanced the quality of the manuscript.

# Reviewer report: Reviewer response for version 1

**DOI:** 10.5256/f1000research.161114.r320045

**Published:** 2024-11-22

**Authors:** Irawan Cosphiadi

**Affiliations:** 1 Division of Hematology and Medical Oncology, Department of Internal Medicine, Faculty of Medicine Universitas Indonesia-Cipto Mangunkusumo National General Hospital, Universitas Indonesia, Jakarta, Indonesia

Dear authors,

 This study presents valuable insights into the role of soluble P-selectin, NET, and MPO as potential risk factors for DVT in cancer patients undergoing platinum-based chemotherapy. The authors have taken a systematic approach to measurement and analysis, employing appropriate case-control matching and utilizing ROC analysis to establish a cut-off for soluble P-selectin, NET, and MPO levels. These methods provide a solid foundation for understanding the biomarker profiles related to thrombotic risk in this population. The thoughtful inclusion of matched controls based on age and gender, along with the detailed measurement procedures, strengthens the study’s contributions to current research in immunothrombosis.

 However, some additional details and analyses could further refine and support the study’s findings. 

1. Despite the role of MPO in promoting oxidative stress and endothelial dysfunction, both of which are implicated in thrombosis, the study notes that MPO levels could not be proven as a risk factor for DVT. The explanation of the measurement methods and use of ROC analysis to determine the MPO cut-off point is helpful. However, further clarification and analysis could strengthen the interpretation of the findings:
  - If possible, detail any cross-reactivity validation of the ELISA kit used (Quantikine® Human MPO Immunoassay) to address concerns about interference from other peroxidases that was mentioned by the authors in the discussion section.
  - Report the AUC, sensitivity, and specificity from the ROC analysis for the chosen MPO cut-off of 644.14 ng/mL.
  - Explore MPO as a continuous variable to offer further insights into its relationship with DVT, potentially revealing associations missed by categorizing it as high/low.
2. It may strengthen the study’s conclusions to include a multivariate analysis, such as logistic regression, to adjust for potential confounders. Cancer type showed a statistically significant difference between cases and controls (p = 0.038), and variables such as type of chemotherapy (p = 0.054) and gender (p = 0.07) had borderline p-values and could be clinically relevant. A multivariate model would help clarify whether these factors independently predict the outcome. Given the sample size, a simple model including only a few key variables (e.g., cancer type, type of chemotherapy, and gender) may be appropriate to avoid overfitting.

Overall, this study makes a valuable contribution to our understanding of thrombotic risk in cancer patients receiving chemotherapy. By incorporating these additional analyses and clarifications, the authors can further enhance the robustness and clinical relevance of their findings.

 Best regards,

 Cosphiadi Irawan

Is the work clearly and accurately presented and does it cite the current literature?

Yes

If applicable, is the statistical analysis and its interpretation appropriate?

Yes

Are all the source data underlying the results available to ensure full reproducibility?

Yes

Is the study design appropriate and is the work technically sound?

Yes

Are the conclusions drawn adequately supported by the results?

Yes

Are sufficient details of methods and analysis provided to allow replication by others?

Partly

Reviewer Expertise:

Hematology and medical oncology

I confirm that I have read this submission and believe that I have an appropriate level of expertise to confirm that it is of an acceptable scientific standard, however I have significant reservations, as outlined above.

# Response

**Published:** 2025-07-05

**Authors:** Ni Made Renny Anggreni Rena

**Affiliations:** Internal Medicine, Udayana University, Jimbaran, Bali, Indonesia

Thank you very much for your constructive feedback. We have added your feedback in the manuscript:

 - Based on the ELISA kit, there are no further cross-reactivity data in human samples, but the kit states that recombinant mouse MPO cross-reacts at approximately 0.4% while neutrophil elastase cross-reacts at approximately 0.25%. We added this to paragraph 4 of the subchapter 'Myeloperoxidase (MPO) as a risk factor for deep vein thrombosis'.

 - We have added the analysis of MPO as a continuous variable into its relationship with DVT in the Result section in Table 4. The data showed that MPO had no significant impact on DVT as numeric data (p=0.588).

 - The AUC, sensitivity, and specificity from the ROC analysis have been added to the narrative of Figure 3 in the Results section.

 - We have included a multivariate logistic regression analysis involving the confounding variables. The results of the analysis are listed in Table 5 of the results section.

 We sincerely acknowledge your constructive input, which has enhanced the quality of the manuscript.

# Reviewer report: Reviewer response for version 1

**DOI:** 10.5256/f1000research.161114.r296985

**Published:** 2024-07-18

**Authors:** Eko Adhi Pangarsa

**Affiliations:** 1 Diponegoro University, Dr. Kariadi General Hospital, Semarang, Indonesia

- In the reference section please add citation author by (Setiawan et al. 2024) (Ref-1)about P-selectin and von Willebrand factor with the risk of DVT in chemotherapy patient published in Thrombosis (Springer Nature) Journal 2020 year. 

 - Please the conclusion of this study suggestion for clinician if the patient has history DVT and undergoing platinum based chemotherapy, please elaborate if possible we change the regimen chemotherapy or add anticoagulant profilaksis.

Is the work clearly and accurately presented and does it cite the current literature?

Partly

If applicable, is the statistical analysis and its interpretation appropriate?

Yes

Are all the source data underlying the results available to ensure full reproducibility?

Yes

Is the study design appropriate and is the work technically sound?

Yes

Are the conclusions drawn adequately supported by the results?

Partly

Are sufficient details of methods and analysis provided to allow replication by others?

Yes

Reviewer Expertise:

Medical Oncology, Hematology (B cell malignancy), Thrombosis Hemostasis

I confirm that I have read this submission and believe that I have an appropriate level of expertise to confirm that it is of an acceptable scientific standard.

## References

[1] : The effectiveness of atorvastatin for the prevention of deep vein thrombosis in cancer patients undergoing chemotherapy : A randomised controlled trial: open label. *Thromb J* .2023;21(1) : 10.1186/s12959-023-00497-0 54 10.1186/s12959-023-00497-0 37150824 PMC10164452

# Response

**Published:** 2025-07-05

**Authors:** Ni Made Renny Anggreni Rena

**Affiliations:** Internal Medicine, Udayana University, Jimbaran, Bali, Indonesia

Thank you very much for your constructive feedback. We have added your feedback in the manuscript.

 - We have included study from Setiawan et al, 2020 which also discusses about P-selectin with the risk of DVT in chemotherapy in paragraph two of the subchapter 'Soluble P-Selectin as a risk factor for deep vein thrombosis'.

 - In the conclusion section, we suggest to the clinicians that the use of anticoagulants as VTE thromboprophylaxis can be considered according to risk stratification based on existing recommendations.
